# Effect of Wnt3a on Keratinocytes Utilizing *in Vitro* and Bioinformatics Analysis

**DOI:** 10.3390/ijms15045472

**Published:** 2014-03-28

**Authors:** Ju-Suk Nam, Chiranjib Chakraborty, Ashish Ranjan Sharma, Young Her, Kee-Jeong Bae, Garima Sharma, George Priya Doss, Sang-Soo Lee, Myung-Sun Hong, Dong-Keun Song

**Affiliations:** 1 Institute for Skeletal Aging & Orthopedic Surgery, Hallym University-Chuncheon Sacred Heart Hospital, Chuncheon 200704, Korea; E-Mails: jsnam88@hallym.ac.kr (J.-S.N.); drchiranjib@yahoo.com (C.C.); boneresearch@hallym.ac.kr (A.R.S.); grant903@gmail.com (K.-J.B.); microbio.garima@gmail.com (G.S.); 123sslee@gmail.com (S.-S.L.); 2 Department of Bio-Informatics, School of Computer and Information Sciences, Galgotias University, Greater Noida 201308, Uttar Pradesh, India; 3 Department of Dermatology, School of Medicine, Kangwon National University Hospital, Chuncheon 200722, Korea; E-Mail: young6078@hanmail.net; 4 Medical Biotechnology Division, School of Biosciences and Technology, VIT University, Vellore 632014, Tamil Nadu, India; E-Mail: georgecp77@yahoo.co.in; 5 Department of Pharmacology, College of Medicine, Hallym University, Chuncheon 200704, Korea

**Keywords:** keratinocyte, tumor necrosis factor, Wnt signaling

## Abstract

Wingless-type (Wnt) signaling proteins participate in various cell developmental processes. A suppressive role of Wnt5a on keratinocyte growth has already been observed. However, the role of other Wnt proteins in proliferation and differentiation of keratinocytes remains unknown. Here, we investigated the effects of the Wnt ligand, Wnt3a, on proliferation and differentiation of keratinocytes. Keratinocytes from normal human skin were cultured and treated with recombinant Wnt3a alone or in combination with the inflammatory cytokine, tumor necrosis factor α (TNFα). Furthermore, using bioinformatics, we analyzed the biochemical parameters, molecular evolution, and protein–protein interaction network for the Wnt family. Application of recombinant Wnt3a showed an anti-proliferative effect on keratinocytes in a dose-dependent manner. After treatment with TNFα, Wnt3a still demonstrated an anti-proliferative effect on human keratinocytes. Exogenous treatment of Wnt3a was unable to alter mRNA expression of differentiation markers of keratinocytes, whereas an altered expression was observed in TNFα-stimulated keratinocytes. *In silico* phylogenetic, biochemical, and protein–protein interaction analysis showed several close relationships among the family members of the Wnt family. Moreover, a close phylogenetic and biochemical similarity was observed between Wnt3a and Wnt5a. Finally, we proposed a hypothetical mechanism to illustrate how the Wnt3a protein may inhibit the process of proliferation in keratinocytes, which would be useful for future researchers.

## 1. Introduction

The Wingless-Type MMTV (mouse mammary tumor virus) integration site family (Wnt) of signaling proteins participates in various developmental process, such as controlling cell proliferation, cell-fate determination, and differentiation during adult homeostasis [1]. Classically, on the basis of their capacity to inhibit phosphorylation of β-catenin by glycogen synthase kinase (GSK)-3β and its subsequent degradation, they are termed as canonical (e.g., Wnt1, 3a, 8), whereas if they do not have any effect on β-catenin levels then they are termed as non-canonical (e.g., Wnt4, 5a, 11) [2,3]. The canonical Wnt ligand, exemplified by Wnt3a, binds to Frizzled (Fz) receptors and co-receptors, low density lipoprotein receptor-related protein-5 (LRP-5), or LRP-6, leading to the activation and nuclear translocation of β-catenin. In the nucleus, β-catenin acts as a transcription factor and governs key cellular processes, such as cell fate determination and stem cell maintenance [4]. By contrast, activation of non-canonical Wnt signaling by ligand like Wnt5a is involved with frizzled (Fz) receptors in conjunction with alternate co-receptors, including ROR1/2 or Ryk, causing β-catenin-independent changes, such as protein kinase C activation and cytoskeletal rearrangements [5]. However, participation of Wnt proteins in the activation of signaling pathways is not as simple as it is presumed. Recent reports on the involvement of Wnt5a [6] and Wnt11 [7] in the activation of β-catenin signaling suggest activation of multiple pathways, depending on which Fz receptors are expressed on the cell surface [3]. In addition, various antagonists of Wnt signaling, such as secreted Frizzled-related proteins (sFRP) [8], Dickkopf (Dkk) proteins [9], Wnt inhibitory factor (WIF)-1 [10], create an extremely complex system of action for Wnt signaling.

Psoriasis is a chronic debilitating disease of the skin characterized by sharply demarcated scaly red plaques commonly located on the extensor surfaces of the skin. The most characteristic feature of psoriasis is chronic inflammation, abnormal proliferation, and differentiation of keratinocytes [11]. The psoriatic epidermis exhibits aberrant expression of antigens associated with hyperproliferation, such as the heterodimer keratin 6–keratin 16 and heat-shock proteins [12]. Moreover, there is some evidence that points to immune activation with lymphocyte infiltration in the psoriatic epidermis and secretion of inflammatory cytokines, such as interleukin (IL)-17, TNFα, and interferon-γ by lymphocytes and dendritic cells [12,13]. Keratinocytes play an important role in the regulation of skin inflammation resulting from environmental and immune cells stimuli. Mitotic activity of basal keratinocytes is increased by a factor of almost 50 in psoriatic skin compared to normal skin, so that keratinocytes only need 3 to 5 days to move from the basal layer to the cornified layer instead of the normal 28 to 30 days [14]. Histological abnormalities in psoriasis have been well documented and include multiple elements that indicate immune-mediated inflammation. However, the cellular and molecular mechanisms underlying the pathogenesis of this alteration in epidermal growth and differentiation remains incompletely understood.

The fundamental roles of most of the Wnt proteins in controlling cell proliferation and differentiation are well established. However, limited information is available about the role of Wnt signaling in hyper-proliferative skin diseases. Nonetheless, global gene expression analysis of psoriasis revealed significant changes in several members of the Wnt ligand family. Notably, non-canonical Wnts like Wnt5a, Wnt16B, and Wnt10A were shown to be up-regulated. Conversely, gene expression profiling of canonical Wnt revealed down-regulation of nearly all members of the canonical Wnt signaling pathway during psoriasis [15]. Of the non-canonical Wnt proteins, Wnt5a was described to have a suppressive effect on normal human keratinocytes growth, while Wnt16b was shown to induce cell proliferation and prolong clonogenicity in primary keratinocytes [15,16]. However, the pathogenic role of canonical Wnt proteins in proliferation and differentiation of keratinocytes is not completely known.

In order to better understand the involvement of canonical Wnt signaling in proliferation and differentiation of keratinocytes, in this study, we performed both experimental research and a series of computational proteomic analyses. The effect of treatment of human primary keratinocytes with the canonical Wnt ligand, Wnt3a, on cell proliferation and differentiation was analyzed. Finally, we used computational methods to determine the relationship between the Wnt family proteins and their conserved regions, the aliphatic index and grand average of hydrophobicity (GRAVY) values, evolution origin, and protein–protein interactions. We corroborated our computational analysis results experimentally by looking at the proliferation and differentiation of keratinocytes. Finally, we concluded that Wnt3a has a significant inhibitory role in the proliferation and differentiation process of keratinocytes and a hypothesis was purported to explain this inhibitory role of Wnt3a in the proliferation and differentiation process of keratinocytes.

## 2. Results

### 2.1. Effect of Wnt3a on the Proliferation of Cultured Keratinocytes

**Figure 1.**
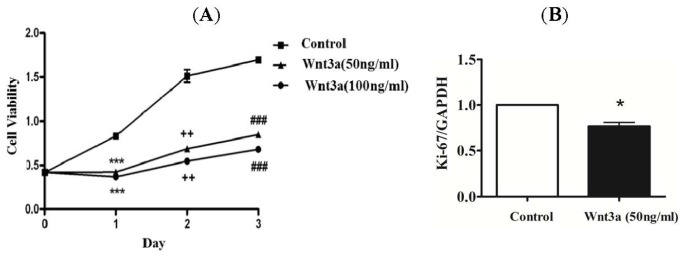
The effect of Wnt3a on the proliferation of keratinocytes. (**A**) Wnt3a suppressed the proliferation of normal keratinocytes in a dose-dependent manner; (**B**) The anti-proliferative effect of Wnt3a on normal keratinocytes was confirmed using real-time PCR of Ki-67. * *p* < 0.05, **^++^** *p* < 0.01, ***^,^**^###^** *p* < 0.001 *vs.* control.

Initially, we attempted to determine whether potentiating canonical Wnt signaling may have any effect on proliferation of human keratinocytes. Treatment of recombinant Wnt3a at a concentration of 50 and 100 ng/mL showed inhibition of keratinocytes proliferation (Figure 1A). The inhibition of proliferating cells was greater at the higher concentration (100 ng/mL) as compared to the lower concentration (50 ng/mL). The anti-proliferative effect of Wnt3a was similarly observed following 1, 2, and 3 days of exposure. Since, expression of the human Ki-67 protein is strictly associated with cell proliferation [17], the anti-proliferative effect of Wnt3a on normal keratinocytes was determined by measuring the expression level of Ki-67 using real-time PCR. The Ki-67 mRNA expression levels confirmed the inhibitory effect of Wnt3a on the proliferation of keratinocytes (Figure 1B).

### 2.2. Effect of Exogenous Wnt3a on the Differentiation of Cultured Keratinocytes

**Figure 2.**
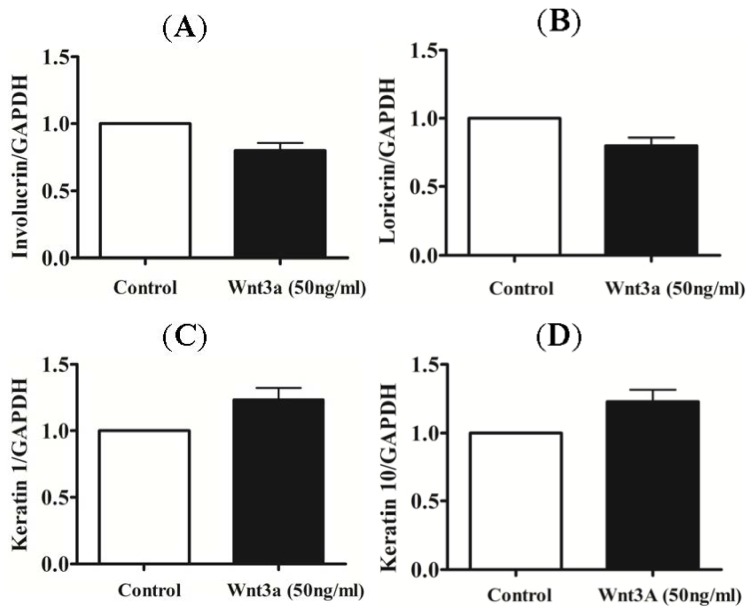
Effect of exogenous Wnt3a on the differentiation process of keratinocytes. Quantitative real-time PCR analysis of early and late differentiation markers for keratinocytes (**A**) involucrin; (**B**) loricrin; (**C**) keratin 1; and (**D**) keratin 10, respectively, showed no significant effect of Wnt3a on the differentiation process of keratinocytes.

In order to further analyze the effect of recombinant Wnt3a on the differentiation process of keratinocytes, total RNA of cultured keratinocytes was isolated after 24 h of treatment with recombinant Wnt3a (50 ng/mL). The mRNA levels of early and late differentiation markers for keratinocytes, including involucrin, keratin 1, loricrin, and keratin 10, were analyzed via real-time PCR. However, no significant change in mRNA levels were observed for any of the markers (Figure 2).

### 2.3. Effect of Wnt3a on Proliferation of TNFprocess of keratinocytes.a

**Figure 3.**
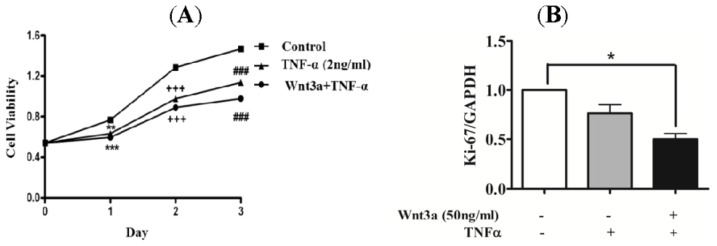
The effect of Wnt3a on the proliferation of TNFα-stimulated keratinocytes. (**A**) Wnt3a suppressed the proliferation of TNFα-stimulated keratinocytes; (**B**) The anti-proliferative effect of Wnt3a on TNFα-stimulated keratinocytes was confirmed using real-time PCR of Ki-67. * *p* < 0.05, ** *p* < 0.01, ***^,^**^+++^**^,^**^###^** *p* < 0.001 *vs.* control.

TNFα is a potent inflammatory cytokine that is highly expressed in psoriatic skin. Moreover, remarkable improvements were observed in clinical trials with TNFα antagonists [18,19] suggesting a crucial role of TNFα in psoriatic pathogenesis. Therefore, to determine the effect of Wnt3a on keratinocyte proliferation under stimulation with TNFα, human primary keratinocytes were treated with TNFα (2 ng/mL) in the absence or presence of Wnt3a (50 ng/mL) (Figure 3). TNFα induced inhibition of proliferating in normal keratinocytes for the observed time period of 1, 2 and 3 days (Figure 3A). Co-treatment with TNFα and Wnt3a significantly suppressed the proliferative ability of keratinocytes as compared to TNFα alone. Furthermore, mRNA expression levels of Ki-67 confirmed this inhibitory effect of TNFα and Wnt3a on keratinocytes (Figure 3B).

### 2.4. Effect of Wnt3a on Differentiation of TNFα-Stimulated Keratinocytes

**Figure 4.**
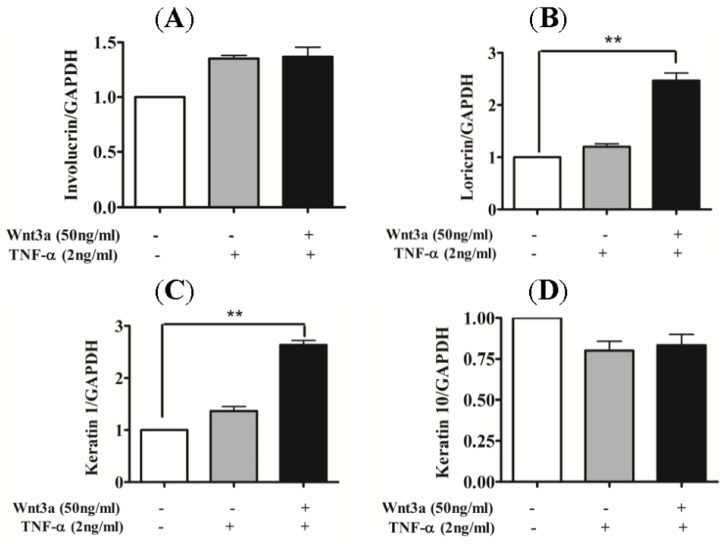
Effect of Wnt3a on the differentiation of TNFα-stimulated keratinocytes. Early differentiation markers, Involucrin (**A**) and keratin 10 (**D**) showed no significant changes under the combinatory treatment of TNFα and Wnt3a, while mRNA levels of the early differentiation marker Keratin 1 (**C**) and late differentiation marker loricrin (**B**) were significantly increased by Wnt3a in TNF stimulated keratinocytes. ** *p* < 0.01 *vs.* control.

In order to evaluate the effect of Wnt3a on the differentiation process of TNFα-stimulated keratinocytes, primary keratinocytes were treated with TNFα (2 ng/mL) with or without Wnt3a for 24 h. Total RNA was collected and mRNA levels of involucrin, keratin 1, loricrin, and keratin 10 were analyzed via real-time PCR. The mRNA expression levels of early and late differentiation markers, keratin 1 and loricrin, showed a 2.5–3.0 fold increase after co-treatment with TNFα and Wnt3a (Figure 4B,C). However, no significant effect of Wnt3a was observed on the expression levels of involucrin and Keratin 10 (Figure 4A,D).

### 2.5. Data Collection of Wnt Family Proteins for Computational Analysis

**Figure 5.**
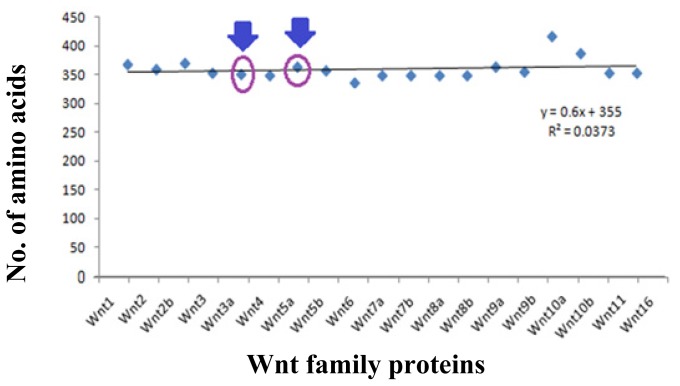
Number of amino acids in the sequence of the various Wnt family member proteins and the trend in their amino acid number. The proteins, Wnt3a and Wnt5a, are marked with an arrow.

Table S1 shows the protein information related to the Wnt family proteins analysed in this study. The protein ID, accession, and version were obtained from the NCBI database. The 19 member proteins of the Wnt family consist of Wnt1, Wnt2, Wnt2b, Wnt3, Wnt3a, Wnt4, Wnt5a, Wnt5b, Wnt6, Wnt7a, Wnt7b, Wnt8a, Wnt8b, Wnt9a, Wnt9b, Wnt10a, Wnt10b, Wnt11, and Wnt16. Figure 5 depicts the number of amino acids present in each protein of the protein family. The highest number of amino acids were determined in Wnt10a (417 aa), whilst the lowest number was found in Wnt6 (338 aa). Specifically, the number of amino acids in Wnt3a and Wnt5a were 352 and 365 amino acids, respectively.

### 2.6. Multiple Sequences Alignment (MSA) of Wnt Proteins

**Figure 6.**
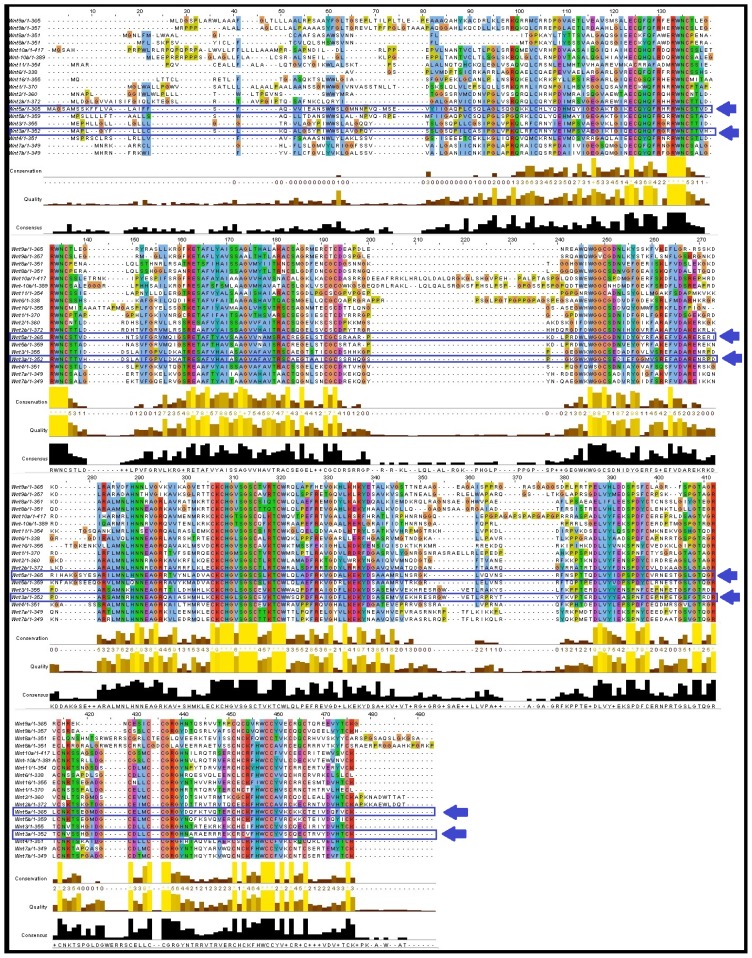
Multiple sequence alignment of the Wnt family member proteins. Wnt3a and Wnt5a align sequence have been marked (arrows) to show the similarity of the sequence. Color codes used for amino acid residues are as follows: A,I,L,M,F,W,V = 
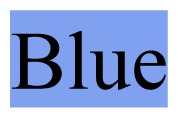
; R,K = 
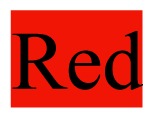
; N,Q,S,T = 
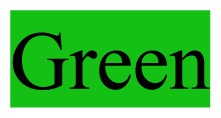
; C = 
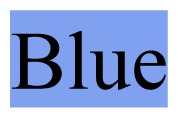
 (Threshhold + 60%); C = 
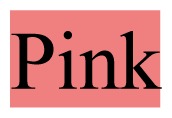
 (Threshhold + 100%); E,D = 
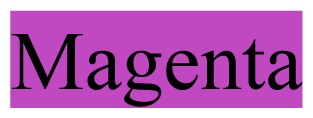
; G = 
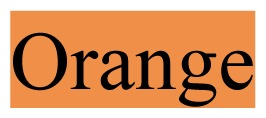
; H,Y = 
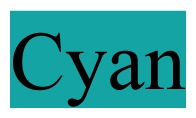
 ; P = 
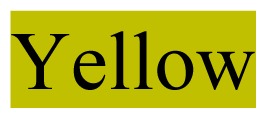
.

Clustal Omega output was visualised through JalView and the results are shown in Figure 6. In total, 16 aligned blocks, including 41, 98–118, 124–130, 132–138, 152–200, 245–270, 282–298, 306–322, 322–346, 380–398, 405–423, 428–433, 436–442, 445–446, and 471–476 were found. The first block is the smallest containing a single residue at the 41st position, whereas the largest aligned block contained 48 residues (152–200). We found that Arg (R), Try (W), Asn (N), Cys (C), Glu (E), Gly (G), Lys (K), His (H), and Ser (S) were well aligned at different positions.

### 2.7. Analysis of the Aliphatic Index and GRAVY of the Wnt Family Proteins

**Figure 7.**
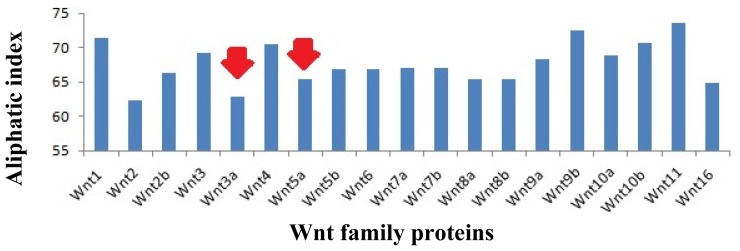
Aliphatic index values of Wnt family member proteins. Wnt3a and Wnt5a aliphatic index have been marked (arrows) to show the resemblance between the values.

The aliphatic side chains are essential for the biological structure and function of proteins, and contain residues, such as alanine, valine, leucine, isoleucine *etc*. The side chains are used to describe the relative volume, called the aliphatic index. Aliphatic hydrophobicity is augmented with an increase in temperature and it is consequently a positive factor to enhance the thermal stability of globular proteins [20]. Our analysis showed the highest aliphatic index values in Wnt11 (73.59). Conversely, the lowest value was noted in Wnt2 (63.33) (Figure 7). In this aliphatic index value scale, a low aliphatic index value was noted for Wnt3a (62.93) and a moderate aliphatic index value for Wnt5a (65.48).

**Figure 8.**
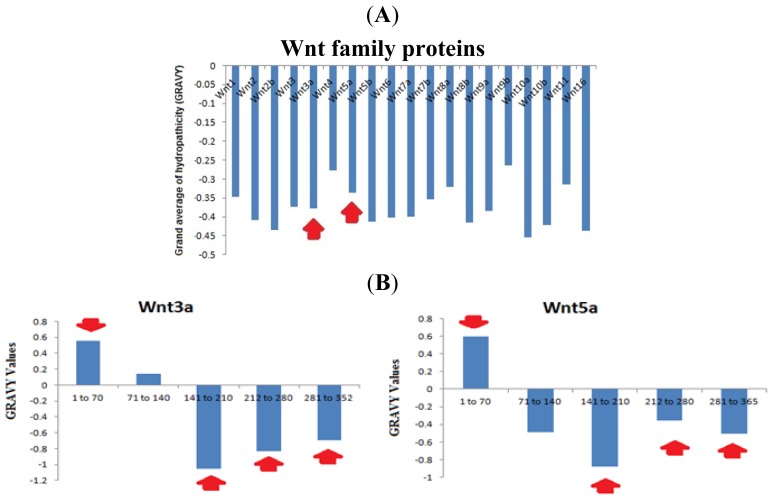
Grand average of hydrophobicity (GRAVY) values of the Wnt family proteins. (**A**) The GRAVY values of 19 proteins of the Wnt family show that all the members of the family are hydrophilic in nature; (**B**) Segmental analysis of GRAVY values of two proteins: Wnt3a and Wnt5a. The analysis shows that the 1st segment (1 to 70 aa) is hydrophobic and the 3rd (141–210), 4th (121–280), and last (281–252 or 281–265) segments are hydrophilic for both proteins. Arrow shows the similarity of the segments between the two proteins.

The grand average of hydrophobicity (GRAVY) is linked with protein solubility. It has been observed that positive GRAVY values are positively associated with hydrophobicity and negatively associated with the hydrophilicity. Hydrophilic proteins form a higher number of hydrogen bonds with water molecules, thereby enhancing the solubility of the protein. The ProtParam GRAVY study predicted that all the Wnt family proteins have negative values (Figure 8A). Our study revealed that the highest negative GRAVY value could be established for Wnt10a (−0.455). Conversely, the lowest negative GRAVY value was determined for Wnt9b (−0.265). After the segment determination of the GRAVY value, we found both hydrophobic and hydrophilic segments in Wnt3a and Wnt5a (Figure 8A). This analysis showed that the 1st segment (1 to 70 aa) is hydrophobic in Wnt3a and Wnt5a. Conversely, the 3rd (141–210), 4th (121–280), and last (281–252 or 281–265) segments are hydrophilic in both Wnt3a and Wnt5a.

### 2.8. Molecular Evolution Analysis of the Wnt Family Proteins

**Figure 9.**
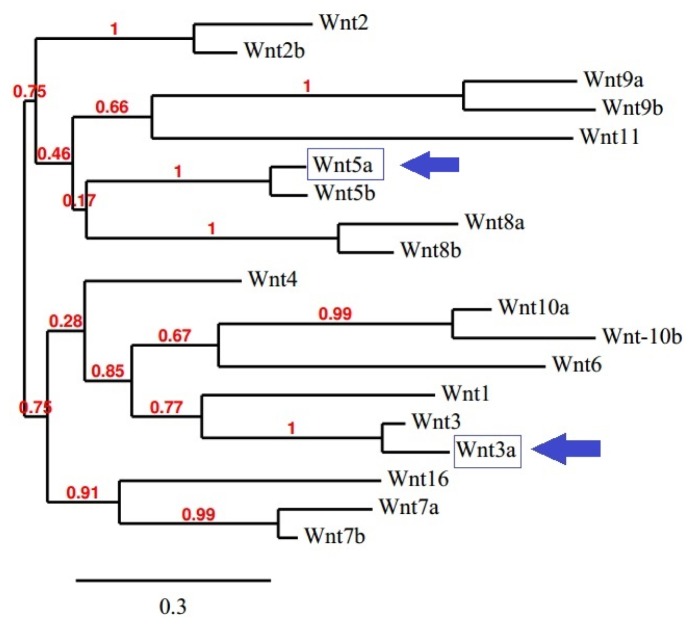
Phylogenetic tree of the 19 members of the Wnt family. Wnt3a and Wnt5a are marked with arrows to show their position.

To understand the molecular evolution of the Wnt family proteins, a phylogenetic tree of the Wnt family proteins was generated and is depicted in Figure 9. The tree illustrates bootstrap values at the inner nodes. It has been established that 5 different groups of proteins have 100% bootstrap replications. We found 100% bootstrap replications between the proteins Wnt2 and Wnt2b; Wnt9a and Wnt9b; Wnt5a and Wnt5b; Wnt8a and Wnt8b; Wnt3 and Wnt3a. The results also showed a 99% bootstrap replication value between Wnt7a and Wnt7b, as well as Wnt10a and Wnt10b.

### 2.9. Creation of the Phylogenetic Tree for the Wnt Family Seed Alignment

**Figure 10.**
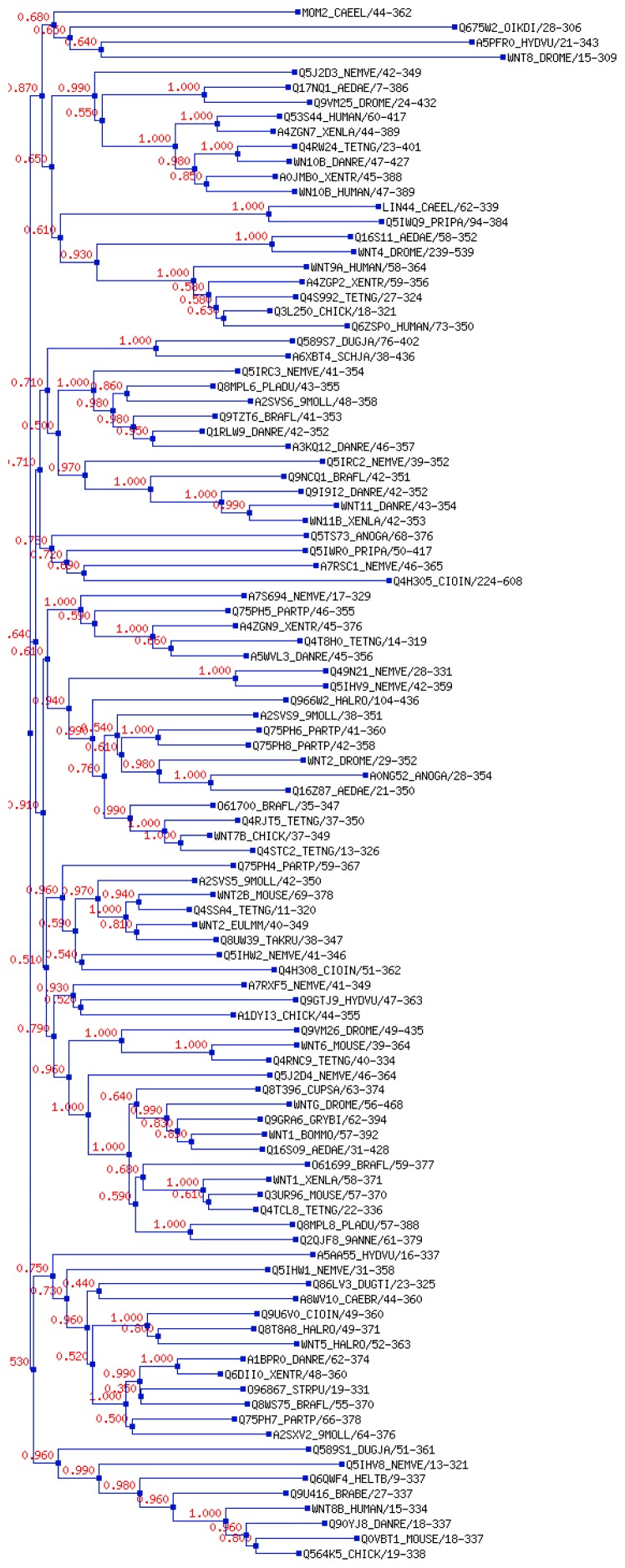
The phylogenetic tree for Wnt family seed alignment utilizing a neighbor join tree algorithm with a local bootstrap based on 100 resamples (which are shown next to the tree nodes).

We also constructed the “phylogenetic tree for family seed alignment” for the Wnt family proteins. It uses sequence similarity across species of Wnt family proteins and portrays the phylogenetic tree for family seed alignment, as shown in Figure 10. The tree shows sequence similarity within 115 different proteins.

### 2.10. Conservation Assessment of Wnt Family Proteins in Different Species

**Figure 11.**
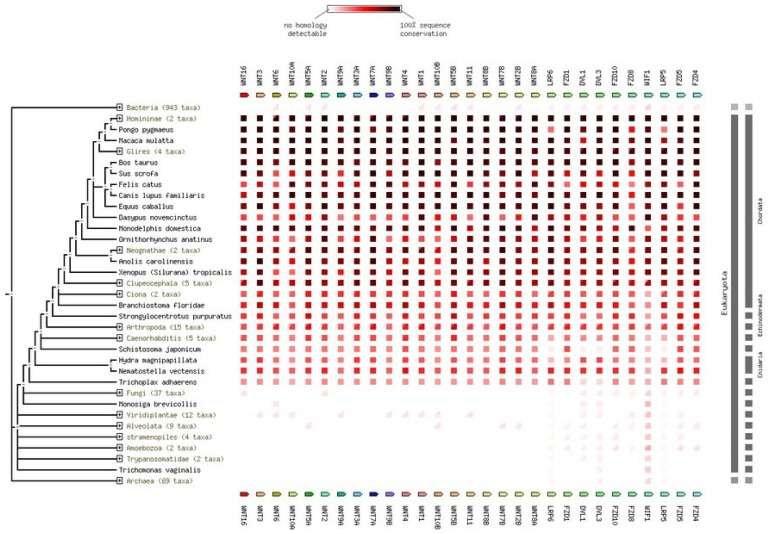
Conservation of Wnt family proteins in different species. The species range includes both eukaryotes and prokaryotes. Conservation of the sequences has been marked with different red color (from lighter red to darker red). For example, lighter red means no detectable homology while darker red means 100% sequence conservation.

The conservation of Wnt family proteins in different species was determined and is documented in Figure 11. These proteins are more conserved in members of the phylum *Chordata*. Other than the phylum *Chordata*, these proteins are also more or less conserved in other families of eukaryotes. The family proteins are not conserved in prokaryotes.

### 2.11. Prediction of Precise Version of Sequence Similarity and Generation of HMM Logos of Wnt Family Proteins

**Figure 12.**
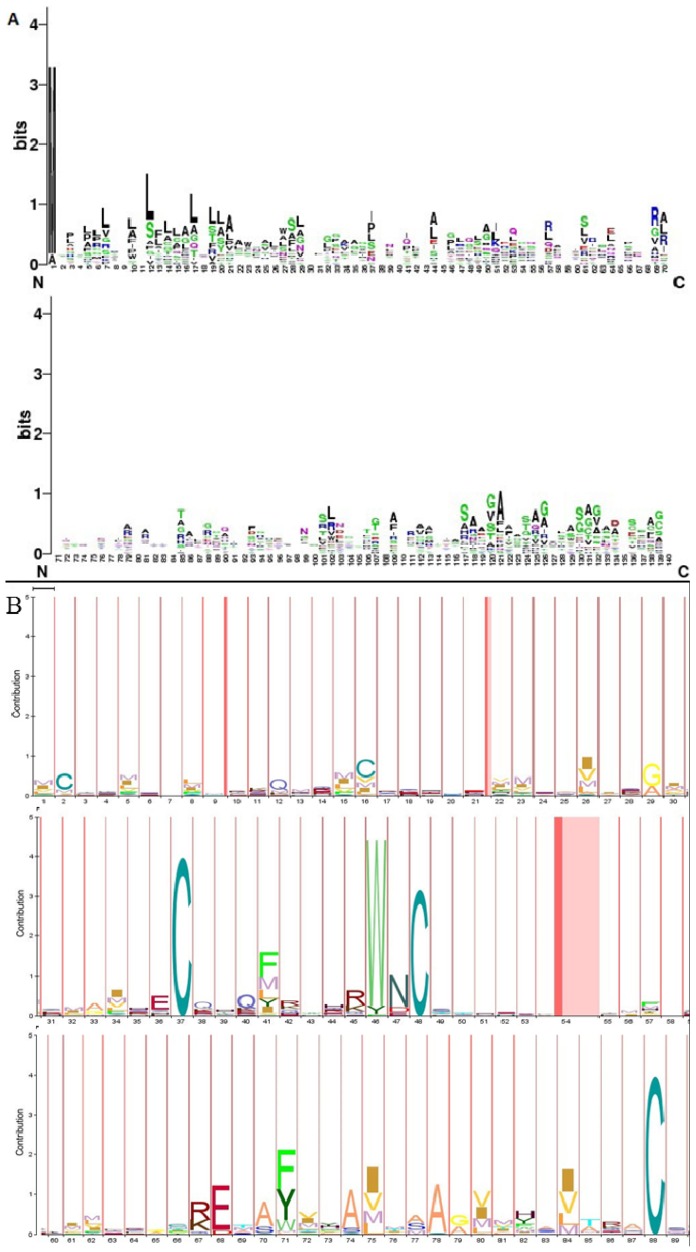
Generated sequence logo of Wnt family proteins. (**A**) Sequence logo up to 140 sequences; (**B**) HMM logo of Wnt family proteins up to 89 sequences. Default colour of webLogo and HMM logo servers has been used.

Two types of logos were created using two different tools, *i.e.*, the sequence logo using WebLogo software and HMM logos using Pfam database. The generated sequence logos are shown in Figure 12A,B. We used 70 amino acids each time to determine the conserved part of the Wnt family proteins. From the sequence logo, we noted that the first sequence logo is the tallest sequence logo (3.4 bits). Methionine (M) may be a highly conserved amino acid in Wnt family proteins at the first position. In addition, we found conserved blocks at 12–17; 18–29; 32–37; 46–55; 60–64; 85–91; 93–96; 99–107; 111–134; 135–139 (Figure 12A). From the HMM logos, we found high sequence logos at various positions, including the 37th (C); 46th (W); 48th (C); 68th (E); 71st (F,Y); 88th (C) (Figure 12B).

### 2.12. Understanding the Protein–Protein Interaction Network of Wnt Family Proteins

**Figure 13.**
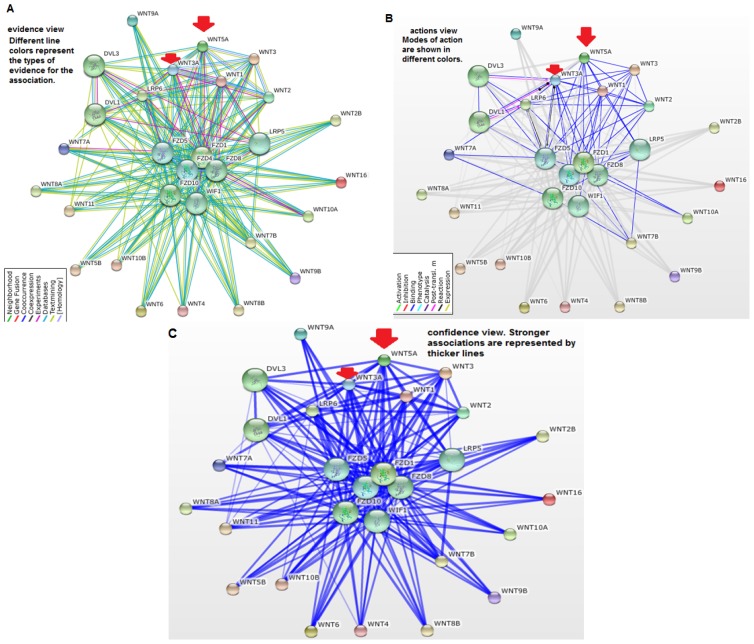
Protein–Protein interaction network of Wnt family proteins using STRING. Arrows shows the similarity in the network between the nodes. (**A**) Network type-evidence network; (**B**) Network type-actions network; (**C**) Network type-confidence network.

The protein–protein network between various Wnt family proteins is depicted in Figure 13. The developed three types of the protein–protein network are shown in Figure 13A (evidence network), Figure 13B (actions network), and Figure 13C (confidence network). The input file for the Wnt family proteins is shown in Figure S1. This figure shows a complex and highly interconnected network between the various Wnt family proteins. From the evidence network, we found that all 19 proteins are closely connected. From the actions network, we determined that binding between proteins is located in the upper position, such as for Wnt3a, Wnt5a, Wnt1, Wnt3, and Wnt2. The network landscape explicates that these proteins are not only interlinked among themselves, but are also interlinked with many other proteins, such as FZD1, FZD4, FZD5, FZD10, WIF1, DVL1, DVL3, LRP5, and LRP6. The confidence network shows that all the proteins are strongly related. An overall stronger association was found between Wnt3a and Wnt5a.

## 3. Discussion

Various skin diseases, especially psoriasis, are characterized by alterations in keratinocyte growth and differentiation [21–23]. In normal skin, the fraction of proliferating keratinocytes is probably around 20%, whereas during psoriasis it is almost 100%. In addition, the mean cell cycle time is reduced from 13 days to 36 h [14]. These changes are associated with altered expression of genes in the epidermis. The late keratinocyte differentiation markers, such as filaggrin and loricrin, are down-regulated, while early differentiation markers, such as involucrin, are up-regulated. Keratinocytes activated by various stressors, including cytokines and growth factors, display a different phenotype (expressing K6, K16 and K17) compared to differentiated cells (expressing K1 and K10) [11]. Recently, TNFα was found to modulate the expression of these proteins via the c-Jun *N*-terminal kinases (JNKs) dependent pathway. It was reported that clinical treatment of psoriasis patients with TNFα antagonists resulted in significant improvement of the epidermal barrier protein expression [14]. As mentioned above, the Wnt family of signaling proteins is a set of highly conserved molecules that participate and control processes, such as cell proliferation, cell-fate differentiation, and differentiation during adult homeostasis [3]. To date, 19 Wnt proteins and 10 Frizzled transmembrane receptors have been documented. Although some knowledge has been accumulated about the state and/or function of Wnt signaling in hyperproliferative skin disorders, very few studies were performed to determine whether and how the Wnt pathway affects keratinocyte proliferation and differentiation [15,24–27]. A recent study, based on microarray interrogation of a large probe set and a large cohort, confirmed that Wnt5a is significantly upregulated in psoriatic lesions [15]. Expression levels of Wnt10A and Wnt7A were found to be moderately increased, while other Wnt members were either not changed or showed decreased expression. Moreover, the study reported a growth-suppressive effect of Wnt5a on human keratinocytes. The effect of the Wnt canonical pathway in keratinocyte biology has been controversial. A recent study showed that R-spondin 2 (Rspo2), a Wnt/β-catenin signaling agonist, is able to increase normal keratinocyte proliferation in a dose-dependent fashion. Rspo2 was found to synergize with Wnt3a to bring about a significant improvement in proliferation [24]. However, another study was unable to observe any effect of recombinant Wnt3a on normal keratinocyte growth and migration [15]. Thus, based on these contradictory reports, it remains unclear whether the canonical pathway has any effect on the proliferation and differentiation of normal keratinocytes. Therefore, in this study, we investigated the effect of exogenous recombinant Wnt3a on the proliferation and differentiation ability of human keratinocytes. Our results indicate that treatment with Wnt3a suppresses the proliferative ability of human keratinocytes (Figure 1A,B), while no effect of Wnt3a treatment was observed on the expression levels of early and late differentiation markers (Figure 2A–D).

TNFα is a potent inflammatory cytokine that is highly expressed in psoriatic skin. This cytokine has a crucial role in psoriasis pathogenesis, as demonstrated by the efficacy of TNFα-targeted therapeutics. TNFα is a powerful inducer of inflammatory gene products in human keratinocytes [28,29]. To investigate the effect of Wnt3a on keratinocytes in psoriatic skin lesions, we induced inflammatory conditions in keratinocytes by treating them with TNFα. TNF receptor signaling has been shown to induce psoriasis-like skin inflammation in keratinocytes [30]. Under these conditions, Wnt3a was able to suppress proliferation of keratinocytes (Figure 3A,B). Taken together, our study results show a suppressive role of Wnt3a on the growth of normal and TNFα-stimulated keratinocytes. Therefore, under the conditions that exist in psoriatic lesions, application of Wnt3a might be applied as an anti-proliferative agent to control the growth of abnormal keratinocytes.

Moreover, Wnt3a treatment increased the expression levels of early and late differentiation markers in TNFα-stimulated keratinocytes, such as keratin 1 and loricrin (Figure 4B,C). Though the expression levels of involucrin and keratin 10 were found to be unaltered, increased expression levels of keratin 1 and loricrin induced by Wnt3a suggest initiation of the differentiation process in TNFα-stimulated keratinocytes. These results imply that Wnt3a may have a recovering effect on the altered differentiation process in keratinocytes, as observed in psoriatic skin lesions.

Our *in silico* analysis showed that the whole Wnt family of signaling proteins are highly conserved. Due to this conserved nature, Wnt proteins are closely associated with vital cellular processes, such as cell signaling, cell-cell communication, and cell fate determination [31,32]. These highly conserved proteins are not only found in humans, but also in other species. Our results indicate that the Wnt family is highly conserved among several members of the phylum *Chordata*, which resembles the Wnt signal-transduction pathway which is widely conserved and may be necessary for important biological process (Figures 10 and 11). The Wnt signaling pathway has not only been found to be conserved in *Chordata,* but even in other species, such as *C. elegans*, *Drosophila etc*. [33,34]. Furthermore, amino acid comparison of the 19 protein members of the Wnt family has shown a homologues range of similarity from 27% to 83% [35]. In our analysis, we also observed high sequence similarity within all Wnt proteins (Figure 6). Sequence similarity between Wnt3a and Wnt5a was also observed in several conserved regions. Conserved residues may be associated with the formation of U-shaped binding contours, which may be necessarily needed to act as a signaling cascade and help in receptor binding [36]. The growth-suppressive effect of Wnt5a on normal human keratinocytes has already been reported [15]. As demonstrated by our *in silico* analysis, a similarity between structural and functional properties of Wnt3a and Wnt5a was observed. Comparable resemblances were observed in the amino acids sequence (Figure 5), multiple sequence alignment (Figure 6), aliphatic index (Figure 7), and GRAVY, as well as segmental GRAVY values (Figure 8). Moreover, we found a strong association between Wnt3a and Wnt5a from the protein–protein interaction networks (evidence network, actions network, and confidence network) during different biological processes (Figure 13). This strongly demonstrates the existence of mutually connected networks between these two proteins. Taken together, the results obtained from *in silico* analysis shows a close relationship between Wnt3a and Wnt5a, and therefore supports our experimental result that Wnt3a is indeed associated with proliferation and differentiation of keratinocytes along with Wnt5a. However, more experimental studies are necessary to fully understand the association between Wnt3a and Wnt5a, as well as to determine the role of the Wnt3a signaling cascade in keratinocyte proliferation and differentiation.

Wnt proteins are not only highly conserved, but their aliphatic index also shows that they are highly stable (Figure 7). Our GRAVY value shows that these proteins are hydrophilic in nature. However, several hydrophobic patches were also detected in these proteins (Figure 8A). The first two segments in Wnt3a were found to be hydrophobic, while the other three were hydrophilic in nature (Figure 8B). Finch *et al.* [37] stated that the signal peptide part of the NH_2_-terminus of a secreted protein contains hydrophobic amino acid segments, which are usually 27 amino acids in number. This observation is quite similar to the pattern of hydrophobic amino acids observed by us, indicating that the first segment of Wnt3a might be acting as a signaling peptide for the protein.

**Figure 14.**
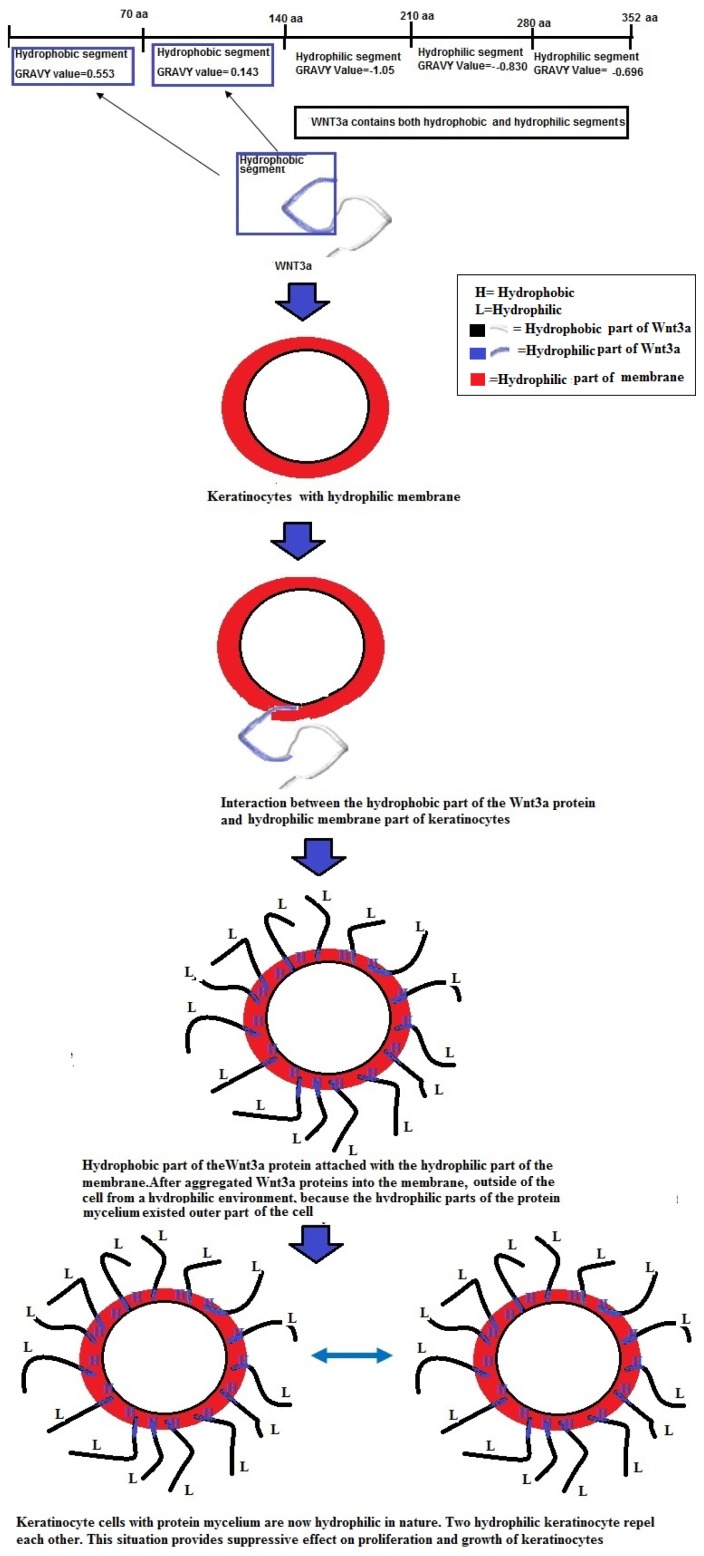
Proposed hypothesis for the interaction of the Wnt3a protein and hydrophilic membrane keratinocytes and how Wnt3a produces the suppressive effect in keratinocytes.

One US patent on human keratinocytes supports the fact that keratinocytes are maintained by hydrophilic membrane [38]. Similar observations on the hydrophilic character of the membrane of human keratinocytes were also reported by Yamato *et al*. [39]. On the basis of the above findings, it may be speculated that the hydrophobic segment present in Wnt3a may help its attachment to the hydrophilic membrane of keratinocytes. It is well established that non-specific hydrophobic associations exist within the plasma membrane. For example, hydrophobic and non-hydrophobic interactions (electrostatic attractions) were reported as a significant event in the interaction of the histactophilin protein with natural hydrophobic anchors [40]. Alternatively, a possible of interaction of the hydrophobic segment of Wnt, acting as signal peptide, with hydrophobic portion (lipid bilayer) of the membrane may be purported. Studies by Gonnet *et al.* have identified hydrophobic proteins attached to cytoplasmic membranes of eukaryotic keratinocytes in complex biological samples [41]. Therefore, we developed a hypothetical structure for illustrating the interaction between the hydrophobic parts of Wnt3a and the hydrophilic membrane of keratinocytes (Figure 14). Here, we tried to depict a proposed mechanism of action to describe how Wnt3a might inhibit the process of proliferation and may contribute to differentiation of keratinocytes. Due to hydrophobic and hydrophilic interactions, Wnt3a proteins might be able to interact with the membrane of keratinocytes making aggregates on its surface and thus creating a hydrophilic environment outside the cell. Previously, hydrophilic repulsion between two biopolymers has been reported through both *in vitro* and *in vivo* experiments [42,43]. Therefore, it is expected that two keratinocytes with aggregated Wnt3a protein on their surfaces would repel each other and thus may contribute to the biological function of keratinocytes, as observed in the experimental results (Figure 13). Nevertheless, our hypothesis is a probable one, but is only possible if the hydrophilic domain contains a net positive or negative charge; the energy needed for repulsion. So, further studies are required to ascertain and understand the biophysical and biochemical interactions between these signaling molecules. Apart from this hypothetical model, activation of the Wnt signaling cascade may also account for suppressed proliferation, both in TNFα treated or untreated keratinocytes, and promote differentiation in TNFα stimulated-keratinocytes. Consequently, to confirm our results and proposed hypothesis and to unfold the pathway of Wnt3a, more studies are needed. These studies should aim to answer different questions, such as (1) whether Wnt3a shows similar effects in psoriatic lesions; (2) determine detailed mechanisms underlying the anti-proliferative effect of Wnt3a on keratinocytes; and (3) elucidate the exact role of the Wnt pathway in the pathogenesis of psoriasis.

## 4. Methods

### 4.1. Tissue Specimens

Primary normal human skin samples were obtained from consented patients who underwent elective surgery at Hallym University Chuncheon Sacred Heart Hospital (Chuncheon, Korea). Sample collections were obtained after patients agreed to donate their skin for research purposes (read and signed the informed consent forms). This study was approved by the Research Ethical Committee of Hallym University, College of Medicine, Chuncheon, Korea.

### 4.2. Cell Culture

Normal human skin samples were washed in saline, minced, and then treated with dispase (Boehringer-Mannheim, Mannheim, Germany) for 4 h at 4 °C. The epidermis was separated and placed in a solution containing 0.025% trypsin (GIBCO-BRL, Grand Island, NY, USA) and 0.01% EDTA at 37 °C for 15 min. After vigorous pipetting, the cells were pelleted and resuspended in serum-free keratinocyte growth medium (KBG gold medium, GIBCO-BRL, Grand-Island, NY, USA) supplemented with bovine pituitary extract, recombinant human epidermal growth factor, insulin, and hydrocortisone. The cells were added to 100 mm dishes (Costar, Pittsburg, NY, USA) and incubated at 37 °C in 5% CO_2_. At 70%–80% confluence, the cells were passaged.

### 4.3. Cell Viability Assay (MTT Assay)

Keratinocytes were cultured in 96-well plates at a concentration of 1 × 10^4^ cells/well for 12 h. Cells were then treated with PBS containing TNF-α (2 ng/mL) or Wnt3a (50 ng/mL; Tocris, Bristol, UK) alone, or in combination in KBG gold medium for 24, 48 and 72 h at 37 °C in 5% CO_2_. Ten micro liters of 3-(4,5-dimethlthiazol-2-yl)-2,5-diphenyl-tetrazoliumbromide (MTT) (5 mg/mL dissolved in PBS; Sigma, St. Lois, MO, USA) was added to each well, and the plates were incubated at 37 °C for 2 h. The supernatant was discarded and 200 μL of dimethyl sulfoxide (DMSO; Sigma, St. Lois, MO, USA) was added to dissolve the blue insoluble MTT formazan produced by mitochondrial succinate dehydrogenase. The absorbance was measured spectrophotometrically at 570 nm. Experiments were repeated three times and the data were expressed as the means ± SD.

### 4.4. Real Time PCR

The cultured keratinocytes were treated with PBS containing TNF-α (2 ng/mL) or Wnt3a (50 ng/mL) alone or in combination in KBG gold medium for 24 h at 37 °C in 5% CO_2_. Total RNA was extracted by using the Trizol reagent (Invitrogen, San Diego, CA, USA) and cDNA was synthesized with 2 μg of total RNA using SuperScript II (Invitrogen, San Diego, CA, USA) according to the manufacturer’s instructions. Real-time PCR was performed using 1 μL of cDNA in a 20 μL reaction volume with the Rotor gene system and QuantiTect SYBR Green PCR Master Mix (Bioneer, Daejeon, Korea). The temperature profile of the reaction was 95 °C for 15 min, 45 cycles of denaturation at 94 °C for 20 s, annealing at 60 °C for 20 s, and extension at 72 °C for 25 s. Primers for keratin 1, keratin 10, involucrin, loricrin, and Ki-67 were obtained from Bioneer (Daejeon, Korea). The forward and reverse primer sequences are listed in Table 1. The relative mRNA levels were normalized by using GAPDH as a housekeeping gene. Rotor gene software was used to compare each gene sample level.

### 4.5. Data Collection of Wnt Family Proteins for Computational Analysis

Computational analysis of Wnt family proteins was performed by obtaining sequence information of human Wnt family proteins from the NCBI database (http://www.ncbi.nlm.nih.gov) [44]. The functional protein sequences were collected in FASTA format along with the accession number from the NCBI database for further analysis.

### 4.6. Multiple Sequences Alignment (MSA) of Wnt Proteins

To understand the resemblance within Wnt proteins, Clustal Omega was used. Clustal Omega server has a graphical edge [45] and utilises several other algorithms, such as “progressive algorithm” and Hidden Markov Models (HMMs) profile to generate alignments. In fact, HMM, is the formal basis for the creation of probabilistic models of linear sequence labelling problems [46,47] and also uses pair wise comparison methods for large-scale sequence analysis. The graphical yield of MSA was visualised through JalView (http://www.jalview.org/).

### 4.7. Analysis of Aliphatic Index and GRAVY of Wnt Family Proteins

A comparison of the various physical and chemical parameters of Wnt family proteins was performed. Two physicochemical parameters, *i.e*., the aliphatic index and grand average of hydrophobicity (GRAVY), were calculated using the ProtParam software from the ExPASy server [48]. In order to understand the segment related hydrophobicity, hydrophilicity and similarity of the two proteins (Wnt3a and Wnt5a), GRAVY values of different segments of the protein chain were determined. Each segment selected for analysis contained 70 amino acids (aa) (1st segment, 1 to 70 aa), (2nd segment, 70 to 140 aa), (3rd segment, 141 to 210 aa), (4th segment, 121 to 280 aa) and last (5th segment, 281–252 or 281–265 aa) of the Wnt3a and Wnt5a proteins.

### 4.8. Molecular Evolution Analysis of Wnt Family Proteins

The molecular evolution was analyzed by developing a phylogenetic tree using the “Phylogeny.fr” server. This server uses several significant algorithms, such as MUSCLE (https://www.ebi.ac.uk/Tools/msa/muscle/) for multiple alignments, PhyML (http://atgc.lirmm.fr/phyml) for tree building, and TreeDyn (http://www.treedyn.org) for tree rendering, as well as G blocks to reconstruct a robust phylogenetic tree from a set of sequences [49]. This also uses the maximum-likelihood method for tree construction along with a Bayesian method for molecular phylogenetics.

### 4.9. Creation of the Phylogenetic Tree for Wnt Family Seed Alignment

To understand the distribution of Wnt family proteins across different species, Pfam database was used [50]. This database contains a large collection of protein families and domain databases, which facilitates the development of a phylogenetic tree for family seed alignment. It utilizes FastTree software, which in turn uses approximately-maximum-likelihood phylogenetic trees. Neighbor join trees were developed using local bootstrap based on 100 resamples to calculate the phylogenetic tree for family seed alignment.

### 4.10. Conservation Assessment of Wnt Family Proteins in Different Species

STRING server was used to understand the conservation of Wnt family proteins in different species [51]. STRING systematically carries out orthology transfers, utilizing two systems at a time: precomputed orthologs from the COG database [52] and a homology-based orthology scheme, computed *de novo* [53]. This server uses more than 7 × 10^11^ pair wise protein comparisons, using the sensitive Smith-Waterman dynamic programming algorithm [54].

### 4.11. Prediction of Precise Version of Sequence Similarity through Sequence Logo and Generation of HMM Logos of Wnt Family Proteins

To understand the more affluent and accurate description of sequence similarity, WebLogo server [55] was used. This tool allows the graphic representation of amino acids and also highlights the patterns in a set of aligned sequences [55]. It has been extensively documented that the sequence logo is at an exact position in the alignment of the residues [56], which can be described as *R*_seq_ according to:

(1) $$R_{\text{seq}}=S_{\text{max}}-S_{\text{obs}}={\text{log}}_{2}\;N-(\sum_{n=1}^{N}p_{\text{n}}\;{\text{log}}_{2}\;p_{\text{n}})$$

where the difference *R*_seq_ is between the maximum possible entropy and the entropy of the observed symbol distribution, *p*_n_ is the observed frequency of symbol *n* at a particular sequence position, and *N* are the number of distinct symbols for the given sequence type.

Presently, the Profile Hidden Markov Model (pHMMs) is a widely used tool for protein family research. In this work, for the generation of HMM logos, we used Pfam database [50], which provides a quick overview of the properties of a HMM in a graphical format [57].

### 4.12. Understanding the Protein–Protein Interaction Network of Wnt Family Proteins

Three types of protein–protein interaction network were developed using the STRING server for the 19 Wnt family proteins: confidence network, evidence network, and actions network. This web-based server investigates possible protein–protein interactions by utilizing direct (physical) and indirect (functional) associations among the members [51].

### 4.13. Statistical Analysis

All the statistical data were analyzed via Graphpad Prism 5.0 (GraphPad Software, San Diego, CA, USA) and evaluated by the two-tailed Student *t* test. Values of *p* < 0.05 were considered to indicate statistical significance.

## 5. Conclusions

Here in, we observed that incubation of human keratinocytes with the canonical Wnt protein, Wnt3a, suppresses the proliferative ability of keratinocytes, while no such conclusive effect was observed on the expression level of differentiation markers. Addition of Wnt3a to TNFα-stimulated keratinocytes also resulted in an anti-proliferative response in keratinocytes. Wnt3a incubation under TNFα-stimulated conditions was able to induce the expression of early and late differentiation markers, implicating Wnt3a in the regulation of the differentiation process under pathogenic conditions. Computational analysis of the Wnt ligand family highlighted biochemical similarities between Wnt3a and Wnt5a. The non-canonical Wnt member, Wnt5a, is a well-known suppressor of keratinocytes proliferation. Moreover, on the basis of the GRAVY value, we were able to deduce a hypothetical model for Wnt3a’s anti-proliferative effect on keratinocytes. Our results and proposed hypothesis might be useful for future researchers to obtain fundamental information about the structural and functional significance relating to the evolutionary biology of Wnt proteins and their role in various disease states, such as psoriasis.

## Supplementary Information



## Figures and Tables

**Table 1.** Primers for real-time PCR.

Target	Forward primer (5′–3′)	Reverse primer (3′–5′)	GenBank No.
*Loricrin*	TAGAGCTCTCATGATGCTACCCGA	GGGTTGGGAGGTAGTTGTACAGAA	NM_000427.2
*Involucrin*	AATGAAACAGCCAACTCCACTGCC	AGGCAGTCCCTTTACAGCAGTCAT	NM_005517.2
*Keratin1*	GGCAGTTCCAGCGTGAAGTTTGTT	TTCTCCGGTAAGGCTGGGACAAAT	NM_006121.3
*Keratin10*	TTGGTGGAGGTAGCTTTCGTGGAA	AGAAGGCCACCATCTCCTCCAAAT	NM_000421.3
*Ki-67*	TGACAAGCCCACGACTGATGAGAA	CTTTGCCTGCTGATGGTGTTCGTT	NM_002417.4
*GAPDH*	TCGACAGTCAGCCGCATCTTCTTT	ACCAAATCCGTTGACTCCGACCTT	NM_002046.3

## References

[1] Kikuchi A., Yamamoto H., Sato A. (2009). Selective activation mechanisms of Wnt signaling pathways. Trends Cell Biol.

[2] Sen M., Ghosh G. (2008). Transcriptional outcome of Wnt-Frizzled signal transduction in inflammation: Evolving concepts. J. Immunol.

[3] Amerongen R.V., Mikels A., Nusse R. (2008). Alternative Wnt signaling is initiated by distinct receptors. Sci. Signal.

[4] Wehrli M., Dougan S.T., Caldwell K., O’Keefe L., Schwartz S., Vaizel-Ohayon D., Schejter E., Tomlinson A., DiNardo S. (2000). Arrow encodes an LDL-receptor-related protein essential for wingless signalling. Nature.

[5] Verkaar F., Zaman G.J.A. (2010). Model for signaling specificity of Wnt/Frizzled combinations through co-receptor recruitment. FEBS Lett.

[6] Liu G., Bafico A., Aaronson S.A. (2005). The mechanism of endogenous receptor activation functionally distinguishes prototype canonical and noncanonical Wnts. Mol. Cell Biol.

[7] Tao Q., Yokota C., Puck H., Kofron M., Birsoy B., Yan D., Asashima M., Wylie C.C., Lin X., Heasman J. (2005). Maternal Wnt11 activates the canonical Wnt signaling pathway required for axis formation in *Xenopus* embryos. Cell.

[8] Kawano Y., Kypta R. (2003). Secreted antagonists of the Wnt signalling pathway. J. Cell Sci.

[9] Logan C.Y., Nusse R. (2004). The Wnt signaling pathway in development and disease. Annu. Rev. Cell Dev. Biol.

[10] Hsieh J.C., Kodjabachian L., Rebbert M.L., Rattner A., Smallwood P.M., Samos C.H., Nusse R., Dawid I.B., Nathans J.A. (1999). New secreted protein that binds to Wnt proteins and inhibits their activities. Nature.

[11] Perera G.K., Di Meglio P., Nestle F.O. (2012). Psoriasis. Annu. Rev. Pathol.

[12] Schon M.P., Boehncke W.H. (2005). Psoriasis. N. Engl. J. Med.

[13] Lebwohl M. (2003). Psoriasis. Lancet.

[14] Weinstein G.D., McCullough J.L., Ross P.A. (1985). Cell kinetic basis for pathophysiology of psoriasis. J. Investig. Dermatol.

[15] Gudjonsson J.E., Johnston A., Stoll S.W., Riblett M.B., Xing X., Kochkodan J.J., Ding J., Nair R.P., Aphale A., Voorhees J.J. (2010). Evidence for altered Wnt signaling in psoriatic skin. J. Investig. Dermatol.

[16] Teh M.T., Blaydon D., Ghali L.R., Briggs V., Edmunds S., Pantazi E., Barnes M.R., Leigh I.M., Kelsell D.P., Philpott M.P. (2007). Role for Wnt16B in human epidermal keratinocyte proliferation and differentiation. J. Cell Sci.

[17] Scholzen T., Gerdes J. (2000). The Ki-67 protein: From the known and the unknown. J. Cell Physiol.

[18] Gordon K.B., Langley R.G., Leonardi C., Toth D., Menter M.A., Kang S., Heffernan M., Miller B., Hamlin R., Lim L. (2006). Clinical response to adalimumab treatment in patients with moderate to severe psoriasis: Double-blind, randomized controlled trial and open-label extension study. J. Am. Acad. Dermatol.

[19] Reich K., Nestle F.O., Papp K., Ortonne J.P., Evans R., Guzzo C., Li S., Dooley L.T., Griffiths C.E., EXPRESS study investigators (2005). Infliximab induction and maintenance therapy for moderate-to-severe psoriasis: A phase III, multicentre, double-blind trial. Lancet.

[20] Ikai A. (1980). Thermostability and aliphatic index of globular proteins. J. Biochem.

[21] Cassano N., Vestita M., Apruzzi D., Vena G.A. (2011). Alcohol, psoriasis, liver disease, and anti-psoriasis drugs. Int. J. Dermatol.

[22] Chamcheu J.C., Siddiqui I.A., Syed D.N., Adhami V.M., Liovic M., Mukhtar H. (2011). *Keratin* gene mutations in disorders of human skin and its appendages. Arch. Biochem. Biophys.

[23] Guttman-Yassky E., Nograles K.E., Krueger J.G. (2011). Contrasting pathogenesis of atopic dermatitis and psoriasis—Part I: Clinical and pathologic concepts. J. Allergy Clin. Immunol.

[24] Chua A.W., Ma D., Gan S.U., Fu Z., Han H.C., Song C., Sabapathy K., Phan T.T. (2011). The role of *R*-spondin2 in keratinocyte proliferation and epidermal thickening in keloid scarring. J. Investig. Dermatol.

[25] Romanowska M., Evans A., Kellock D., Bray S.E., McLean K., Donandt S., Foerster J. (2009). Wnt5a exhibits layer-specific expression in adult skin, is upregulated in psoriasis, and synergizes with type 1 interferon. PLoS One.

[26] Yamaguchi Y., Passeron T., Hoashi T., Watabe H., Rouzaud F., Yasumoto K., Hara T., Tohyama C., Katayama I., Miki T. (2008). Dickkopf 1 (DKK1) regulates skin pigmentation and thickness by affecting Wnt/beta-catenin signaling in keratinocytes. FASEB J.

[27] Reischl J., Schwenke S., Beekman J.M., Mrowietz U., Sturzebecher S., Heubach J.F. (2007). Increased expression of Wnt5a in psoriatic plaques. J. Investig. Dermatol.

[28] Chiricozzi A., Guttman-Yassky E., Suarez-Farinas M., Nograles K.E., Tian S., Cardinale I., Chimenti S., Krueger J.G. (2011). Integrative responses to IL-17 and TNF-α in human keratinocytes account for key inflammatory pathogenic circuits in psoriasis. J. Investig. Dermatol.

[29] Fujiwara S., Nagai H., Oniki S., Yoshimoto T., Nishigori C. (2012). Interleukin (IL)-17 *vs.* IL-27: opposite effects on tumor necrosis factor-α-mediated chemokine production in human keratinocytes. Exp. Dermatol.

[30] Kumari S., Bonnet M.C., Ulvmar M.H., Wolk K., Karagianni N., Witte E., Uthoff-Hachenberg C., Renauld J.C., Kollias G., Toftgard R. (2013). Tumor necrosis factor receptor signaling in keratinocytes triggers interleukin-24-dependent psoriasis-like skin inflammation in mice. Immunity.

[31] Nusse R., Varmus H. (2012). Three decades of Wnts: A personal perspective on how a scientific field developed. EMBO J.

[32] MacDonald B.T., Tamai K., He X. (2009). Wnt/β-catenin signaling: Components, mechanisms, and diseases. Dev. Cell.

[33] Cadigan K.M., Nusse R. (1997). Wnt signaling: A common theme in animal development. Genes Dev.

[34] Kumar M., Ahmad S., Ahmad E., Saifi M.A., Khan R.H. (2012). *In silico* prediction and analysis of Caenorhabditis EF-hand containing proteins. PLoS One.

[35] Miller J. (2002). The Wnts. Genome Biol.

[36] Ain Q.U., Seemab U., Rashid S., Nawaz M.S., Kamal M.A. (2013). Prediction of structure of human Wnt-CRD (FZD) complex for computational drug repurposing. PLoS One.

[37] Finch P.W., He X., Kelley M.J., Uren A., Schaudies R.P., Popescu N.C., Rudikoff S., Aaronson S.A., Varmus H.E., Rubin J.S. (1997). Purification and molecular cloning of a secreted, Frizzled-related antagonist of Wnt action. Proc. Natl. Acad. Sci. USA.

[38] Hansbrough J.F. (1997). U.S. Human Keratinocytes Supported on a Hydrophilic Membrane and Methods of Using Same to Effect Wound Closure. U.S. Patent.

[39] Yamato M., Utsumi M., Kushida A., Konno C., Kikuchi A., Okano T. (2001). Thermo-responsive culture dishes allow the intact harvest of multilayered keratinocyte sheets without dispase by reducing temperature. Tissue Eng.

[40] Hanakam F., Gerisch G., Lotz S., Alt T., Seelig A. (1996). Binding of hisactophilin I and II to lipid membranes is controlled by a pH-dependent myristoyl-histidine switch. Biochemistry.

[41] Gonnet F., Lemaitre G., Waksman G., Tortajada J. (2003). MALDI/MS peptide mass fingerprinting for proteome analysis: Identification of hydrophobic proteins attached to eucaryote keratinocyte cytoplasmic membrane using different matrices in concert. Proteome Sci.

[42] Van Oss C.J. (2003). Long-range and short-range mechanisms of hydrophobic attraction and hydrophilic repulsion in specific and aspecific interactions. J. Mol. Recognit.

[43] Kundu S.K., Gupta S., Stellbrink J., Willner L., Richter D. (2013). Relating structure and flow of soft colloids. Eur. Phys. J. Spec. Top.

[44] Sayers E.W., Barrett T., Benson D.A., Bolton E., Bryant S.H., Canese K., Chetvernin V., Church D.M., DiCuccio M., Federhen S. (2011). Database resources of the national center for biotechnology information. Nucleic Acids Res.

[45] Sievers F., Wilm A., Dineen D., Gibson T.J., Karplus K., Li W., Lopez R., McWilliam H., Remmert M., Söding J. (2011). Fast, scalable generation of high-quality protein multiple sequence alignments using Clustal Omega. Mol. Syst. Biol.

[46] Eddy S.R. (1998). Profile hidden Markov models. Bioinformatics.

[47] Eddy S.R. (2004). What is a hidden Markov model?. Nat. Biotechnol.

[48] Gasteiger E., Gattiker A., Hoogland C., Ivanyi I., Appel R.D., Bairoch A. (2003). ExPASy: The proteomics server for in-depth protein knowledge and analysis. Nucleic Acids Res.

[49] Dereeper A., Guignon V., Blanc G., Audic S., Buffet S., Chevenet F., Dufayard J.F., Guindon S., Lefort V., Lescot M. (2008). Phylogeny.fr: Robust phylogenetic analysis for the non-specialist. Nucleic Acids Res.

[50] Punta M., Coggill P.C., Eberhardt R.Y., Mistry J., Tate J., Boursnell C., Pang N., Forslund K., Ceric G., Clements J. (2012). The Pfam protein families database. Nucleic Acids Res.

[51] Jensen L.J., Kuhn M., Stark M., Chaffron S., Creevey C., Muller J., Doerks T., Julien P., Roth A., Simonovic M. (2009). STRING 8—A global view on proteins and their functional interactions in 630 organisms. Nucleic Acids Res.

[52] Tatusov R.L., Fedorova N.D., Jackson J.D., Jacobs A.R., Kiryutin B., Koonin E.V., Krylov D.M., Mazumder R., Mekhedov S.L., Nikolskaya A.N. (2003). The COG database: An updated version includes eukaryotes. BMC Bioinform.

[53] Mering C.V., Jensen L.J., Snel B., Hooper S.D., Krupp M., Foglierini M., Jouffre N., Huynen M.A., Bork P. (2005). STRING: known and predicted protein–protein associations, integrated and transferred across organisms. Nucleic Acids Res.

[54] Smith T.F., Waterman M.S. (1981). Identification of common molecular subsequences. J. Mol. Biol.

[55] Crooks G.E., Hon G., Chandonia J.M., Brenner S.E. (2004). WebLogo: A sequence logo generator. Genome Res.

[56] Schneider T.D., Stephens R.M. (1990). Sequence logos a new way to display consensus sequences. Nucleic Acids Res.

[57] Schuster-Bocklerm B., Schultz J., Rahmann S. (2004). HMM Logos for visualization of protein families. BMC Bioinform.

