# Supplementary material for: Effect of Wnt3a on Keratinocytes Utilizing in Vitro and Bioinformatics Analysis
Source: Int J Mol Sci. 2014 Mar 28;15(4):5472–95. doi: 10.3390/ijms15045472 (PMC4013576; doi:10.3390/ijms15045472)
Supplement: Supplementary file 1 [file ijms-15-05472-s001.pdf]

Supplementary Information

Table S1. Functional proteins associated Wnt proteins (Homo sapiens) and their protein IDs analysed in this study.

Seq No.	Protein	Protein ID	Other Information
1	Wnt1	NCBI Reference Sequence: NP_005421.1	DEFINITION: proto-oncogene Wnt-1 precursor [Homo sapiens]; ACCESSION: NP_005421; VERSION: NP_005421.1 GI:4885655
2	Wnt2	NCBI Reference Sequence: NP_003382.1	DEFINITION: protein Wnt-2 precursor [Homo sapiens]; ACCESSION: NP_003382; VERSION: NP_003382.1 GI:4507927
3	Wnt2B	NCBI Reference Sequence: NP_004176.2	DEFINITION protein Wnt-2b isoform WNT-2B1 [Homo sapiens]; ACCESSION: NP_004176; VERSION: NP_004176.2 GI:13518017
4	Wnt3	NCBI Reference Sequence: NP_110380.1	DEFINITION proto-oncogene Wnt-3 precursor [Homo sapiens]; ACCESSION: NP_110380; VERSION: NP_110380.1 GI:13540477
5	Wnt3A	NCBI Reference Sequence: NP_149122.1	DEFINITION protein Wnt-3a precursor [Homo sapiens]; ACCESSION: NP_149122; VERSION: NP_149122.1 GI:14916475
6	Wnt4	NCBI Reference Sequence: NP_110388.2	DEFINITION protein Wnt-4 precursor [Homo sapiens]; ACCESSION: NP_110388; VERSION: NP_110388.2 GI:174029
7	Wnt5A	NCBI Reference Sequence: NP_001243034.1	DEFINITION protein Wnt-5a isoform 2 precursor [Homo sapiens]; ACCESSION: NP_001243034; VERSION: NP_001243034.1 GI:371502087
8	Wnt5B	NCBI Reference Sequence: NP_110402.2	DEFINITION protein Wnt-5b precursor [Homo sapiens]; ACCESSION: NP_110402; VERSION: NP_110402.2 GI:17402919
9	Wnt6	GenBank: AAG45154.1	DEFINITION WNT6, partial [Homo sapiens]; ACCESSION: AAG45154; VERSION: AAG45154.1 GI:1200735
10	Wnt7A	NCBI Reference Sequence: NP_004616.2	DEFINITION protein Wnt-7a precursor [Homo sapiens]; ACCESSION NP_004616; VERSION: NP_004616.2 GI:17505191;
11	Wnt7B	NCBI Reference Sequence: NP_478679.1	DEFINITION protein Wnt-7b precursor [Homo sapiens]; ACCESSION NP_478679 XP_001718810; VERSION: NP_478679.1 GI:17505193;
12	Wnt8A	GenBank: BAB60960.1	DEFINITION WNT8A [Homo sapiens]; ACCESSION BAB60960; VERSION: BAB60960.1 GI:14495176
13	Wnt8B	NCBI Reference Sequence: NP_003384.2	DEFINITION protein Wnt-8b precursor [Homo sapiens]; ACCESSION NP_003384; VERSION: NP_003384.2 GI:110735437
14	Wnt9A	NCBI Reference Sequence: NP_003386.1	DEFINITION protein Wnt-9a precursor [Homo sapiens]; ACCESSION NP_003386; VERSION: NP_003386.1 GI:15082261

Table S1. *Cont.*

Seq No.	Protein	Protein ID	Other Information
15	Wnt9B	GenBank: AAQ88584	DEFINITION WNT9B [Homo sapiens]; ACCESSION AAQ88584; VERSION: AAQ88584.1 GI:37181546
16	Wnt10A	NCBI Reference Sequence: NP_079492.2	DEFINITION protein Wnt-10a precursor [Homo sapiens]; ACCESSION NP_079492; VERSION: NP_079492.2 GI:16936520
17	Wnt10B	NCBI Reference Sequence: NP_003385.2	DEFINITION protein Wnt-10b precursor [Homo sapiens]; ACCESSION NP_003385; VERSION: NP_003385.2 GI:16936522
18	Wnt11	NCBI Reference Sequence: NP_004617.2	DEFINITION protein Wnt-11 precursor [Homo sapiens]; ACCESSION NP_004617; VERSION: NP_004617.2 GI:17017974
19	Wnt16	NCBI Reference Sequence: NP_057171.2	DEFINITION protein Wnt-16 isoform 2 [Homo sapiens]; ACCESSION NP_057171; VERSION: NP_057171.2 GI:17402914

Figure S1. Input file for network development using string.

Your Input:

WNT16	wingless-type MMTV integration site family, member 16; Ligand for members of the frizzled family of seven transmembrane receptors. Probable developmental protein. May be a signaling molecule which affects the development of discrete regions of tissues. Is likely to signal over only few cell diameters (By similarity) (365 aa)
WNT3	wingless-type MMTV integration site family, member 3; Ligand for members of the frizzled family of seven transmembrane receptors. Wnt-3 and Wnt-3a play distinct roles in cell-cell signaling during morphogenesis of the developing neural tube (By similarity) (355 aa)
WNT6	wingless-type MMTV integration site family, member 6; Ligand for members of the frizzled family of seven transmembrane receptors. Probable developmental protein. May be a signaling molecule which affects the development of discrete regions of tissues. Is likely to signal over only few cell diameters (365 aa)
WNT10A	wingless-type MMTV integration site family, member 10A; Ligand for members of the frizzled family of seven transmembrane receptors. Probable developmental protein. May be a signaling molecule important in CNS development. Is likely to signal over only few cell diameters (417 aa)
WNT5A	wingless-type MMTV integration site family, member 5A; Ligand for members of the frizzled family of seven transmembrane receptors. Can activate or inhibit canonical Wnt signaling, depending on receptor context. In the presence of FZD4, activates beta-catenin signaling. In the presence of ROR2, inhibits the canonical Wnt pathway by promoting beta-catenin degradation through a GSK3-independent pathway which involves down-regulation of beta-catenin-induced reporter gene expression. Suppression of the canonical pathway allows chondrogenesis to occur and inhibits tumor formation. Stimulates [...] (380 aa)
WNT2	wingless-type MMTV integration site family member 2; Ligand for members of the frizzled family of seven transmembrane receptors. Probable developmental protein. May be a signaling molecule which affects the development of discrete regions of tissues. Is likely to signal over only few cell diameters (360 aa)
WNT9A	wingless-type MMTV integration site family, member 9A; Ligand for members of the frizzled family of seven transmembrane receptors. Probable developmental protein. May be a signaling molecule which affects the development of discrete regions of tissues. Is likely to signal over only few cell diameters (By similarity) (365 aa)
WNT3A	wingless-type MMTV integration site family, member 3A; Ligand for members of the frizzled family of seven transmembrane receptors. Wnt-3 and Wnt-3a play distinct roles in cell-cell signaling during morphogenesis of the developing neural tube (352 aa)
WNT7A	wingless-type MMTV integration site family, member 7A; Ligand for members of the frizzled family of seven transmembrane receptors. Probable developmental protein. Signaling by Wnt-7a allows sexually dimorphic development of the mullerian ducts (By similarity) (349 aa)
WNT9B	wingless-type MMTV integration site family, member 9B; Ligand for members of the frizzled family of seven transmembrane receptors. Probable developmental protein. May be a signaling molecule which affects the development of discrete regions of tissues. Is likely to signal over only few cell diameters (By similarity) (357 aa)
WNT9B	wingless-type MMTV integration site family, member 9B; Ligand for members of the frizzled family of seven transmembrane receptors. Probable developmental protein. May be a signaling molecule which affects the development of discrete regions of tissues. Is likely to signal over only few cell diameters (By similarity) (357 aa)
WNT4	wingless-type MMTV integration site family, member 4; Ligand for members of the frizzled family of seven transmembrane receptors. Probable developmental protein. May be a signaling molecule which affects the development of discrete regions of tissues. Is likely to signal over only few cell diameters (By similarity). Overexpression may be associated with abnormal proliferation in human breast tissue (351 aa)
WNT1	wingless-type MMTV integration site family, member 1; Ligand for members of the frizzled family of seven transmembrane receptors. Probable developmental protein. May be a signaling molecule important in CNS development. Is likely to signal over only few cell diameters (370 aa)
WNT10B	wingless-type MMTV integration site family, member 10B; Ligand for members of the frizzled family of seven transmembrane receptors. Probable developmental protein. May be a signaling molecule which affects the development of discrete regions of tissues. Is likely to signal over only few cell diameters (By similarity) (389 aa)
WNT5B	wingless-type MMTV integration site family, member 5B; Ligand for members of the frizzled family of seven transmembrane receptors. Probable developmental protein. May be a signaling molecule which affects the development of discrete regions of tissues. Is likely to signal over only few cell diameters (By similarity) (359 aa)
WNT11	wingless-type MMTV integration site family, member 11; Ligand for members of the frizzled family of seven transmembrane receptors. Probable developmental protein. May be a signaling molecule which affects the development of discrete regions of tissues. Is likely to signal over only few cell diameters (354 aa)
WNT8B	wingless-type MMTV integration site family, member 8B; Ligand for members of the frizzled family of seven transmembrane receptors. May play an important role in the development and differentiation of certain forebrain structures, notably the hippocampus (351 aa)
WNT7B	wingless-type MMTV integration site family, member 7B; Ligand for members of the frizzled family of seven transmembrane receptors. Probable developmental protein. May be a signaling molecule which affects the development of discrete regions of tissues. Is likely to signal over only few cell diameters (By similarity) (349 aa)
WNT2B	wingless-type MMTV integration site family, member 2B; Ligand for members of the frizzled family of seven transmembrane receptors. Probable developmental protein. May be a signaling molecule which affects the development of discrete regions of tissues. Is likely to signal over only few cell diameters. May be involved in normal development or differentiation as well as in carcinogenesis (391 aa)
WNT8A	wingless-type MMTV integration site family, member 8A; Ligand for members of the frizzled family of seven transmembrane receptors. May play an important role in the development and differentiation of certain forebrain structures, notably the hippocampus (351 aa)

(Homo sapiens)
